# Transcriptome Analysis and Discovery of Genes Involved in Immune Pathways from Hepatopancreas of Microbial Challenged Mitten Crab *Eriocheir sinensis*

**DOI:** 10.1371/journal.pone.0068233

**Published:** 2013-07-17

**Authors:** Xihong Li, Zhaoxia Cui, Yuan Liu, Chengwen Song, Guohui Shi

**Affiliations:** 1 Key Laboratory of Experimental Marine Biology, Institute of Oceanology, Chinese Academy of Sciences, Qingdao, China; 2 Graduate University of the Chinese Academy of Sciences, Beijing, China; 3 National & Local Joint Engineering Laboratory for Ecological Mariculture, Institute of Oceanology, Chinese Academy of Sciences, Qingdao, China; Temasek Life Sciences Laboratory, Singapore

## Abstract

**Background:**

The Chinese mitten crab *Eriocheir sinensis* is an important economic crustacean and has been seriously attacked by various diseases, which requires more and more information for immune relevant genes on genome background. Recently, high-throughput RNA sequencing (RNA-seq) technology provides a powerful and efficient method for transcript analysis and immune gene discovery.

**Methods/Principal Findings:**

A cDNA library from hepatopancreas of *E. sinensis* challenged by a mixture of three pathogen strains (Gram-positive bacteria *Micrococcus luteus*, Gram-negative bacteria *Vibrio alginolyticus* and fungi *Pichia pastoris*; 10^8^ cfu·mL^−1^) was constructed and randomly sequenced using Illumina technique. Totally 39.76 million clean reads were assembled to 70,300 unigenes. After ruling out short-length and low-quality sequences, 52,074 non-redundant unigenes were compared to public databases for homology searching and 17,617 of them showed high similarity to sequences in NCBI non-redundant protein (Nr) database. For function classification and pathway assignment, 18,734 (36.00%) unigenes were categorized to three Gene Ontology (GO) categories, 12,243 (23.51%) were classified to 25 Clusters of Orthologous Groups (COG), and 8,983 (17.25%) were assigned to six Kyoto Encyclopedia of Genes and Genomes (KEGG) pathways. Potentially, 24, 14, 47 and 132 unigenes were characterized to be involved in Toll, IMD, JAK-STAT and MAPK pathways, respectively.

**Conclusions/Significance:**

This is the first systematical transcriptome analysis of components relating to innate immune pathways in *E. sinensis*. Functional genes and putative pathways identified here will contribute to better understand immune system and prevent various diseases in crab.

## Introduction

Chinese mitten crab *Eriocheir sinensis* (Crustacea: Decapoda: Grapsidae, Eriocheir) (Henri Milne Edwards, 1854) is one of the important economic aquaculture species in China. However, with rapid development of large-scale culture, frequent outbreaks of diseases caused by viruses, bacteria and rickettsia-like organisms have led to catastrophic economic losses in cultured *E. sinensis* stocks [1]–[3]. Characterizing immune molecules and understanding defense mechanism are useful to health management and disease control in crab aquaculture.

Like other invertebrates, *E. sinensis* lacks adaptive immune system and mainly depends on innate immunity. Innate immune system provides a first line for host to defense against invading pathogens. It is composed of cellular responses like phagocytosis and encapsulation, and humoral responses that produce immune-related factors. Immune relevant genes, such as crustin [4], cathepsin L [5], prophenoloxidase (proPO) [6], C-type lectin [7] and anti-lipopolysaccharide factor (ALF) [8], have been separately cloned and characterized from *E. sinensis*. However, knowledge about immune system of *E. sinensis* is still fragmentary and different signaling pathways implicated in immune response also remain incomplete.

To date, genome sequence of any crab species is still unavailable, which limits resources of molecular information. In recent years, high-throughput RNA-sequencing (RNA-Seq), including Solexa/Illumina, Roche/454 and ABI/SOLiD, has offered high-effective technology for analysis of gene expression, discovery of novel transcripts, identification of differentially expressed genes and others [9]. The powerful technology provides a new opportunity for studies of genome reference-free species and non-model organisms. With development of this technology, RNA-Seq has been widely applied in various invertebrates, such as *Eriocheir sinensis* [10], [11], *Litopenaeus vannamei* [12], *Bactrocera dorsalis* [13], *Pinctada martensii* [14] and *Crassostrea gigas* [15].

In crustacean, apart from functioning as a digestive gland, hepatopancreas is also an important immune organ that functions as a primary site to synthesize and excrete immune molecules, such as beta-1,3-glucan binding protein (LGBP) [16], antibacterial peptide (AMP) [17], lectin or lectin related proteins and others [18]. Expressed sequence tag (EST) analysis and gene discovery of *L. vannamei* and *L. setiferus* also demonstrated that hepatopancreas played a crucial role in innate immunity and hepatopancreas cDNA library appeared to be more diverse than hemocytes library [18]. Hence, large-scale identification of immune genes from hepatopancreas is of great value and necessity to study immune mechanism in crustacean. Previously, Jiang et al [19] constructed a nonnormalized hepatopancreas cDNA library of *E. sinensis* and characterized immune-associated genes by EST approach. It aided to understand biological function of hepatopancreas and served a basis for in-depth investigation of Chinese mitten crab.

In the present study, by Illumina sequencing and bioinformatics analysis, we analyzed hepatopancreas transcriptome of *E. sinensis* that was infected with a mixture of three pathogen strains (Gram-positive bacteria *Micrococcus luteus*, Gram-negative bacteria *Vibrio alginolyticus* and fungi *Pichia pastoris*; 10^8^ cfu·mL^−1^). Main objective of this study was to annotate functional genes from this transcriptome analysis and identify potential immune molecules of different signaling pathways, such as Toll, immune deficiency (IMD), janus kinase (JAK)-signal transducers and activators of transcription (STAT) and mitogen-activated protein kinase (MAPK) pathways.

## Materials and Methods

### Ethic Statment

This study was strictly performed in accordance with the Guide for Care and Use of Laboratory Animals by Chinese Association for Laboratory Animal Sciences (No. 2011-2).

### Mitten crab and microbial challenge

Healthy mature female mitten crabs were obtained from a commercial farm in Panjin, China and acclimatized in oxygenated seawater at 15±1°C for a week before processing. During whole period of the experiment, all crabs were fed with clam meat and the water was changed every day. For immune challenge experiment, we prepared a mixture of three pathogen strains (Gram-positive bacteria *Micrococcus luteus*, Gram-negative bacteria *Vibrio alginolyticus* and fungi *Pichia pastoris*), which were suspended in 0.1 mol/L PBS (pH 7.0) with the final pathogens concentration of 10^8^ cfu·mL^−1^. The crabs were injected at arthrodial membrane of the last walking leg with 100 μL the mixture of pathogens and returned into seawater tanks for 8 h. Hepatopancreas of treated crabs were collected and kept in liquid nitrogen for RNA extraction.

### RNA isolation and cDNA library construction

Total RNA was isolated with Trizol Reagent (Invitrogen), after which the concentration, quality and integrity were determined with a NanoDrop spectrophotometer and an Agilent 2100 Bioanalyzer. Poly-(A)-containing mRNA was purified using oligo(dT) magnetic beads and Oligotex mRNA Kits (Qiagen). The mRNA was fragmented and used as template to synthesize first-stranded cDNA with reverse transcriptase and random hexamer-primers. Second-stranded cDNA was synthesized using RNase H and DNA polymerase I. These double-stranded cDNA fragments underwent process of end repair, addition of a single ‘A’ base and ligation of adapters. Adaptor modified fragments were selected by gel purification and amplified through PCR to create the final cDNA library.

### Illumina sequencing, assembly, and annotation

Transcriptome sequencing was carried out on an Illumina HiSeq 2000 platform that generated about 100 bp paired-end (PE) raw reads (Novogene Bioinformatics Technology Co.Ltd). Raw sequences were deposited to NCBI Short Read Archive (SRA) database (http://www.ncbi.nlm.nih.gov/Traces/sra/). After removing adaptor sequences, ambiguous ‘N’ nucleotides (with the ratio of ‘N’ to be more than 10%) and low quality sequences (with quality score to be less than 5), the remaining clean reads were assembled using Trinity software as described for de novo transcriptome assembly without reference genome [20].

For homology annotation, non-redundant sequences were subjected to public databases including NCBI (http://www.ncbi.nlm.nih.gov/) non-redundant protein (Nr) and non-redundant nucleotide (Nt), Swiss-Prot (http://www.ebi.ac.uk/uniprot/), Gene Ontology (GO) (http://www.geneontology.org/), Clusters of Orthologous Groups (COG) (http://www.ncbi.nlm.nih.gov/COG/) and Kyoto Encyclopedia of Genes and Genomes (KEGG) (http://www.genome.jp/kegg/). If results of different databases were conflicted, a priority order of alignments from Nr, Nt, KEGG, Swiss-Prot, GO and COG databases was followed. Comparing to Nr, Nt and Swiss-Prot databases was carried out using BlastX algorithm with an E-value cut-off of 10^−10^. GO terms at 2^nd^ level was used to perform GO annotation. COG and KEGG classification were done using BlastX with an E-value cut-off of 10^−5^.

### Immune gene identification

Immune genes belonging to different signaling pathways were manually identified according to annotated sequences in above databases. Protein coding sequences (CDSs) were also predicted by Trinity software and multiple sequence alignment was carried out using ClustalX.

### Gene expression validation

Genes identified in this transcriptome sequencing analysis were validated and quantified by real-time PCR (RT-PCR). Primers (Table S1) were designed according to Illumina sequencing data with Primer Premier 5. Prepared total RNA used in RT-PCR analysis was isolated from the same treated crab hepatopancreas as that in Illumina sequencing. Reversed cDNA was also synthesized using the same method as described in Illumina sequencing preparation.

RT-PCR was performed in an ABI 7300 Real-time Detection System (Applied Biosystems). β-actin of *E. sinensis* was used as an internal control to normalize the expression level and all experiments were performed in triplicate. The reaction was carried out in a total volume of 10 μL, containing 5 μL of 2× SYBR Premix Ex Taq^TM^ II (TaKaRa), 0.2 μL of 50× ROX Reference Dye, 2 μL of diluted cDNA mix, 0.2 μL of each primer (10 mM) and 2.4 μL of Milli-Q water. Thermal profile for SYBR Green RT-PCR was 95°C for 5 min, followed by 40 cycles of 95°C for 5 s and 60°C for 31 s. To confirm that only one PCR product was amplified and detected, dissociation curve analysis of amplification products was performed at the end of each PCR reaction. After the PCR program, data were analyzed with ABI 7300 SDS software (Applied Biosystems). The comparative CT method (2^−ΔΔ^ CT method) was used to analyze the expression level of different genes.

## Results

### Transcriptome sequencing and assembly

**Figure 1:**
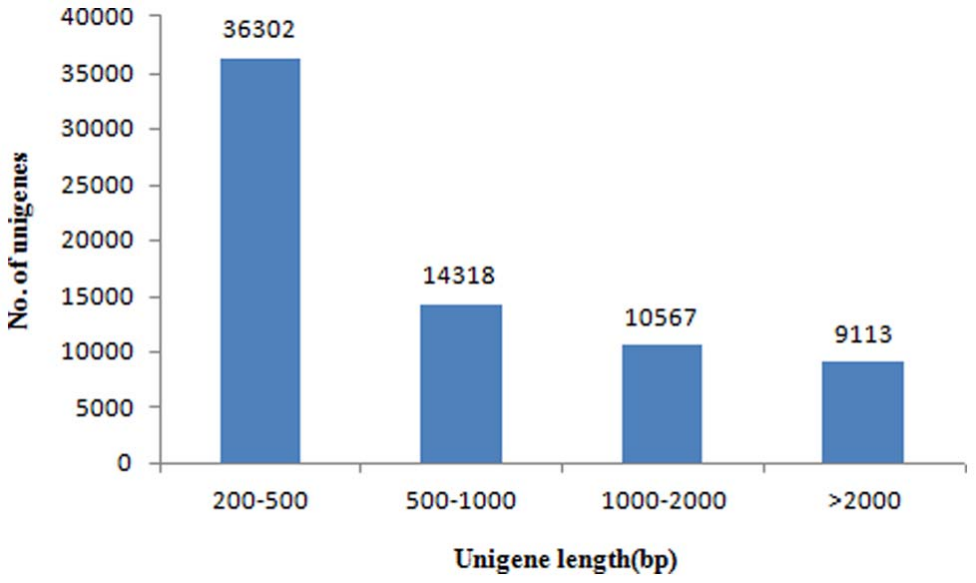
Length dirstribution of assembled unigenes.

Illumina sequencing data from microbial challenged *E. sinensis* hepatopancreas were deposited to NCBI SRA database under accession number of SRA068878. Approximately 40.78 million Illumina PE raw reads were generated (Table 1). After removing adaptor sequences, ambiguous nucleotides and low-quality sequences, 39.76 million clean reads with an average length of 101.10 bp remained. Assembly of clean reads resulted in 70,300 unigenes that ranged from 201 bp to 16874 bp with a N50 length of 1834 bp (Table 1). Length statistics of assembled unigenes were displayed (Figure 1).

**Table 1:** Summary of sequences analysis.

Description	Number
**Before trimming**	
Raw reads	40.78×10^6^
**After trimming**	
Clean reads	39.76×10^6^
Clean bases (Mb)	4.02×10^3^
Average length of clean reads (bp)	101.10
GC content (%)	46.01
Q20 percentage (%)	97.95
**After assembly**	
Unigenes	70,300
Min length (bp)	201
Max length (bp)	16,874
Average length (bp)	967
N50 (bp)	1,834
N90 (bp)	354

### Blast analysis

**Figure 2:**
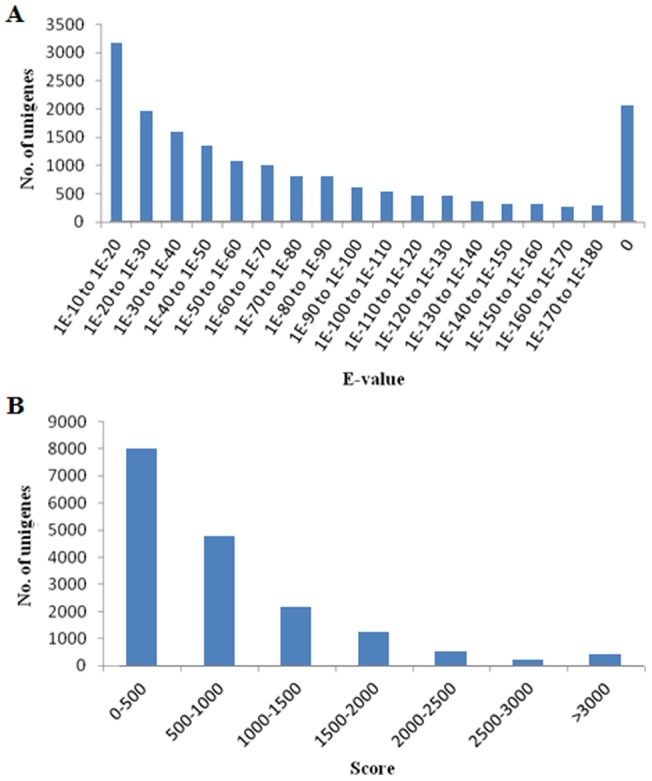
E-value and score distribution of unigenes matched to Nr database. (A) E-value distribution of annotated unigenes. (B) Score distribution of annotated unigenes.

After eliminating repeated and short-length sequences, 52,074 non-redundant unigenes were subjected to public databases for similarity searching. 17,617 (33.83%) and 5,033 (9.67%) non-redundant unigenes (Table 2) showed identity with sequences in NCBI Nr and Nt databases, respectively. E-value and score distribution of best hits in Nr database revealed that 55.45% (9,481) of matched sequences showed high homology with an E-value <1E-50 and 55.79% (9,539) with a score >500 (Figure 2). Our results also showed that 28.54% (14,862) of non-redundant unigenes demonstrated similarity to known genes in Swiss-Prot database (Table 2).

**Table 2:** BLAST analysis of non-redundant unigenes against public databases.

Database	Number of annotated unigenes	Percentage of annoted unigenes
**Nr**	17,617	33.83%
**Nt**	5,033	9.67%
**Swiss-Prot**	14,862	28.54%
**KEGG**	8,983	17.25%
**GO**	18,734	36.00%
**COG**	12,243	23.51%

### Functional annotation and pathway assignment

**Figure 3:**
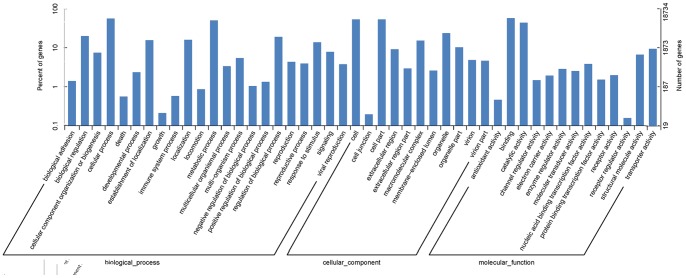
GO categorization of non-redundant unigenes. Each annotated sequence was assigned at least one GO term.

According to Gene Ontology (GO), an international standardized gene functional classification system, 18,734 non-redundant unigenes were classified into three major functional categories (biological process, cellular component and molecular function) and 46 subcategories (Figure 3). In the category of biological process, dominant subcategories were ‘cellular process’ (10,321, 23.84%) and ‘metabolic process’ (9,268, 21.41%). Of sequences categorized as cellular component, ‘cell’ (9,864, 29.69%) and ‘cell part’ (9,864, 29.69%) were most represented, followed by ‘organelle’ (4,315, 13.08%) and ‘macromolecular complex’ (2,776, 8.36%). Among molecular function terms, they showed a significant proportion of clusters assigned to ‘binding’ (10,562, 42.78%) and ‘catalytic activity’ (8,086, 32.75%). However, within each of the three categories, few genes were assigned to subcategories of ‘growth’, ‘cell junction’ and ‘receptor regulator activity’.

**Figure 4:**
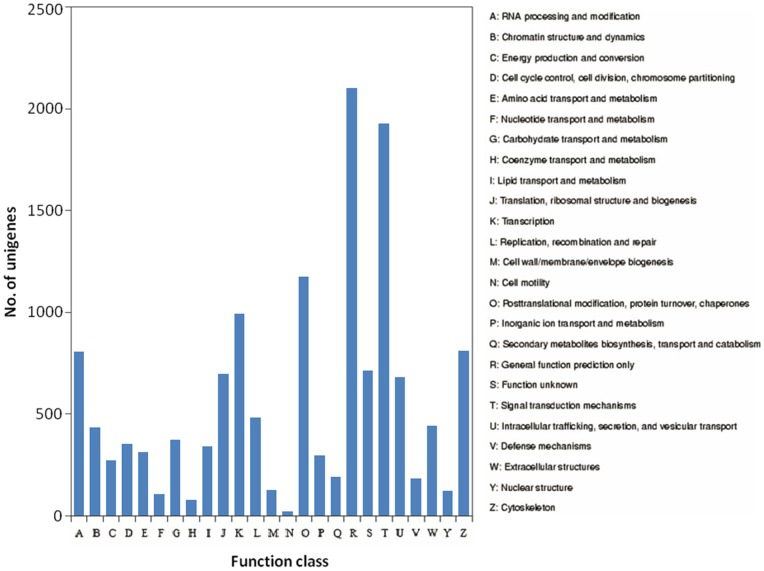
COG annotation of putative proteins.

To classify orthologous gene products, 12,243 (23.51%) non-redundant unigenes (Table 2) were subdivided into 25 COG classifications. Among them, the cluster of ‘general function prediction only’ (2,086, 14.92%) represented the largest group, followed by ‘signal transduction mechanisms’ (1,913, 13.68%), ‘post-translational modification, protein turnover, chaperon’ (1,167, 8.35%) and ‘transcription’ (984, 7.04%), whereas ‘cell mobility’ (23, 0.16%) was the smallest group (Figure 4).

Using KEGG, 8983 unigenes (Table 2) were assigned to six specific pathways, including metabolism, cellular processes, organism system, human diseases, genetic information processing and environmental information processing (Table 3). Totally 2,582 unigenes were identified in metabolism and main metabolism terms were ‘carbohydrate metabolisms’, ‘nucleotide metabolisms’ and ‘amino acid metabolisms’. Dominant subcategories of other five pathways were ‘cell growth and death’, ‘nervous system’, ‘infectious diseases’, ‘translation’ and ‘signal transduction’, respectively.

**Table 3:** KEGG classification of non-redundant unigenes.

KEGG category	KEGG subcategory	No. of unigenes
**Metabolism**	Amino acid metabolism	368
	Biosynthesis of other secondary metabolites	59
	Carbohydrate metabolism	513
	Energy metabolism	302
	Glycan biosynthesis and metabolism	247
	Lipid metabolism	300
	Metabolism of cofactors and vitamins	149
	Metabolism of other amino acids	120
	Metabolism of terpenoids and polyketides	48
	Nucleotide metabolism	381
	Xenobiotics biodegradation and metabolism	95
**Genetic information**	Folding	629
**processing**	Replication and repair	523
	Transcription	294
	Translation	707
**Environmental**	Membrane transport	31
**information processing**	Signal transduction	776
	Signaling molecules and interaction	96
**Cellular**	Cell communication	282
**processes**	Cell growth and death	597
	Cell motility	90
	Transport and catabolism	466
**Organismal**	Circulatory system	68
**systems**	Development	116
	Digestive system	264
	Endocrine system	339
	Environmental adaptation	48
	Excretory system	126
	Immune system	418
	Nervous system	478
	Sensory system	53
**Human diseases**	Cancers	782
	Cardiovascular diseases	111
	Endocrine and metabolic diseases	22
	Immune diseases	82
	Infectious diseases	1,366
	Neurodegenerative diseases	443
	Substance dependence	149

### Immune gene and pathway analysis

High-throughput sequencing effort revealed that a large number of molecules were highly enriched in immune processes and signaling pathways. Among them, we focused on key genes involved in Toll, IMD, JAK-STAT and MAPK signaling pathways. Main components of these immune pathways were described as follow.

#### Toll pathway

**Figure 5:**
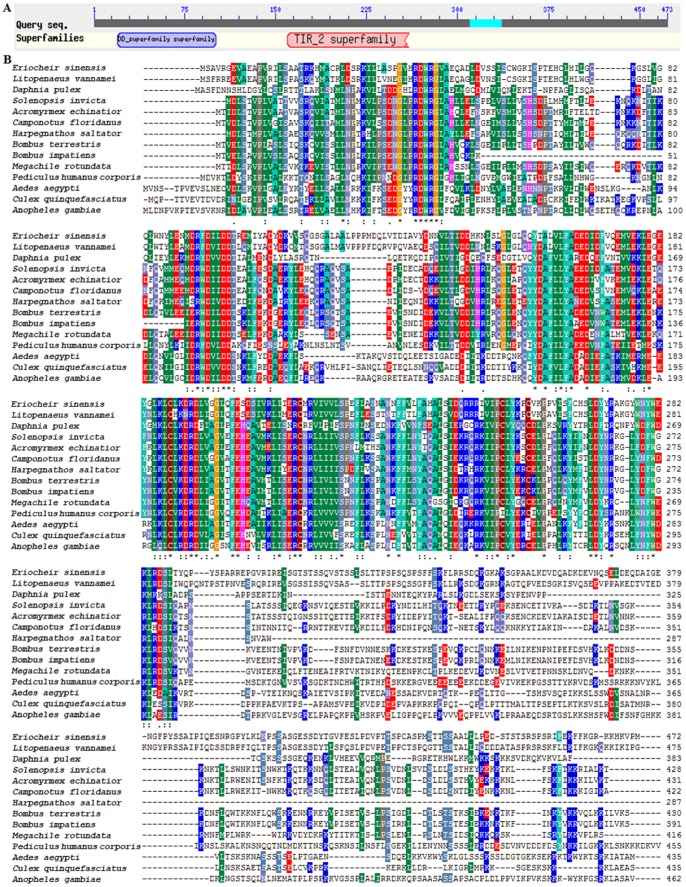
Predicted domain and multiple sequence alignment of MyD88. (A) Putative conserved domain of *E. sinensis* MyD88. (B) Multiple sequence alignment of *E. sinensis* MyD88 with homologs from other thirteen organisms. Species and GenBank accession numbers of other MyD88 sequences were as follow: *Litopenaeus vannamei* (AFP49300.1), *Camponotus floridanus* (EFN62977.1), *Pediculus humanus corporis* (XP_002431564.1), *Solenopsis invicta* (EFZ13196.1), *Acromyrmex echinatior* (EGI65212.1), *Harpegnathos saltator* (EFN82696.1), *Bombus terrestris* (XP_003394201.1), *Aedes aegypti* (XP_001658635.1), *Megachile rotundata* (XP_003705811.1), *Bombus impatiens* (XP_003489479.1), *Daphnia pulex* (EFX88460.1), *Anopheles gambiae* (XP_314167.4), *Culex quinquefasciatus* (XP_001868621.1).

Twenty-six non-redundant unigenes were identified with identity to main molecules of Toll pathway, including Spatzle, Toll, myeloid differentiation factor 88 (MyD88), Pelle, Cactus, Dorsal/ Dorsal-related immunity factor (Dif) (Table 4, Figure S1). Tube and tumor necrosis factor receptor-associated factor 6 (TRAF6) appeared to be absent in this study. In putative Toll pathway, microbial components triggered activating of Spaetzle and Toll, which initiated signaling pathway by recruiting MyD88 and other molecules (Tube, Pelle and TRAF6). Then, it induced nuclear translocation of Dorsal/Dif. The key adaptor protein coordinating Toll pathway, MyD88, contained a death domain (DD) and a Toll/interleukin-1 receptor (TIR) domain (Figure 5A). Multiple sequence alignment of MyD88 from *E. sinensis* and other thirteen species revealed that they were more similar at N-terminus and less conserved at C-terminus (Figure 5B). Sequence of *E. sinensis* MyD88 showed highest identity (65%) to homolog from *L. vannamei*.

**Table 4:** Putative immune genes involved in Toll pathway.

Signaling molecular	Unigene	ID	E-value	Description
**Spatzle**	comp20879_c1	AEL23015.1	2.97E-32	protein spaetzle [*Cherax quadricarinatus*]
	comp20655_c0	ACD36030.1	6.58E-61	spatzle protein [*Fenneropenaeus chinensis*]
	comp30857_c1	ACD36030.1	6.58E-61	spatzle protein [*Fenneropenaeus chinensis*]
**Toll**	comp283944_c0	XP_971999.1	0	PREDICTED: similar to toll [*Tribolium castaneum*]
	comp318091_c0	XP_971999.1	0	PREDICTED: similar to toll [*Tribolium castaneum*]
	comp318804_c0	XP_971999.1	0	PREDICTED: similar to toll [*Tribolium castaneum*]
	comp401835_c0	XP_971999.1	0	PREDICTED: similar to toll [*Tribolium castaneum*]
	comp6372_c0	XP_971999.1	0	PREDICTED: similar to toll [*Tribolium castaneum*]
	comp30733_c0	XP_003209739.1	5.18E-43	PREDICTED: toll-like receptor 13-like [*Meleagris gallopavo*]
	comp15258_c0	BAF99007.1	0	toll receptor [*Marsupenaeus japonicas*]
	comp18754_c0	BAF99007.1	1.33E-42	toll receptor [*Marsupenaeus japonicas*]
	comp2035_c0	BAF99007.1	0	toll receptor [*Marsupenaeus japonicas*]
	comp27278_c0	BAF99007.1	0	toll receptor [*Marsupenaeus japonicas*]
	comp27278_c1	BAF99007.1	1.33E-42	toll receptor [*Marsupenaeus japonicas*]
	comp24095_c0	ABK88278.1	3.33E-17	toll-like receptor [*Carcinoscorpius rotundicauda*]
**MyD88**	comp23475_c0	EFN62977.1	1.53E-52	Myeloid differentiation primary response protein MyD88 [*Camponotus floridanus*]
**Pelle**	comp30621_c0	JN180645.1	4.34E-13	*Litopenaeus vannamei* pelle mRNA, complete cds [*Litopenaeus vannamei*]
	comp29278_c0	XP_002431275.1	2.65E-11	conserved hypothetical protein [*Pediculus humanus corporis*]
**Cactus**	comp400958_c0	XP_001927565.2	3.02E-47	PREDICTED: tonsoku-like protein-like isoform 1 [*Sus scrofa*]
**Dorsal/Dif**	comp115581_c0	ACZ98167.1	0	dorsal [*Litopenaeus vanname*]
	comp1171_c0	ACZ98167.1	0	dorsal [*Litopenaeus vanname*]
	comp15051_c0	ACZ98167.1	0	dorsal [*Litopenaeus vanname*]
	comp28273_c0	ADM14334.1	7.09E-178	short gastrulation protein [*Parhyale hawaiensis*]
	comp415679_c0	ADM14334.1	7.09E-178	short gastrulation protein [*Parhyale hawaiensis*]

#### IMD pathway

Fourteen non-redundant unigenes showed similarity to signaling molecules of IMD pathway, such as IMD, transforming growth factor beta–activated kinase (dTAK1), inhibitor of nuclear factor kappa-B kinase (IKK), Dredd and Relish, while Fas-associated death domain protein (dFADD) was not detected (Table 5, Figure S2). In putative IMD pathway, bacterial components activated the adaptor protein IMD, causing signaling cascade and finally leading to activation of Relish. Relish then regulated expression of antimicrobial peptide (AMP) and other immune-related genes.

**Table 5:** Putative immune genes involved in IMD pathway.

Signaling molecular	Unigene	ID	E-value	Description
**IMD**	comp14472_c0	ACL37048.1	3.54E-25	IMD [*Litopenaeus vanname*]
	comp18071_c0	ACL37048.1	3.54E-25	IMD [*Litopenaeus vanname*]
**dTAK1**	comp27862_c0	XP_002408296.1	1.02E-21	mitogen activated protein kinase kinase kinase 1, MAPKKK1, MEKK1, putative [*Ixodes scapularis*]
	comp10992_c0	EFN79813.1	1.13E-92	Mitogen-activated protein kinase kinase kinase 7 [*Harpegnathos saltator*]
**IKK**	comp27376_c0	AAC05683.1	5.60E-56	I-kappa-B kinase [*Crassostrea gigas*]
	comp17756_c0	AAX56336.1	3.37E-90	ikk-like protein [*Pinctada fucata*]
**Dredd/Casp**	comp3072_c0	ADH94025.1	5.84E-55	caspase [*Marsupenaeus japonicus*]
	comp19217_c0	ADH94015.1	8.26E-63	caspase [*Marsupenaeus japonicas*]
	comp10698_c0	ADM45311.1	3.38E-27	caspase [*Eriocheir sinensis*]
	comp298860_c0	ADM45311.1	6.08E-32	caspase [*Eriocheir sinensis*]
	comp16422_c0	XP_003385047.1	1.33E-20	PREDICTED: caspase-3-like [*Amphimedon queenslandica*]
	comp419824_c0	XP_003385047.1	1.33E-20	PREDICTED: caspase-3-like [*Amphimedon queenslandica*]
**Relish**	comp27894_c0	ADM14334.1	1.03E-168	relish [*Eriocheir sinensis*]
	comp29150_c7	ADM14334.1	0	relish [*Eriocheir sinensis*]

#### JAK-STAT pathway

Various molecules involved in JAK-STAT signaling pathway were characterized in our analysis (Table 6, Figure S3). Through JAK-STAT pathway, different aspects of hematopoiesis and immune response were mediated by cytokines, including interleukin (IL), interferon (IFN), growth hormone (GH) and thyroid peroxidase (TPO) [21]. In putative JAK-STAT pathway, after interaction of cytokine and cytokine receptor (CytokineR), STAT was activated by JAK, dimerizing, translocating to the nucleus and regulating the expression of target genes. Furthermore, numerous regulatory layers were found in this pathway. They were divided to negative regulators including SH2-containing phosphatases SHP1 and SHP2, cytokine inducible SH2-containing protein (CIS), suppressor of cytokine signaling (SOCS) and protein inhibitor of activated STAT (PIAS), and positive regulators including signal transducing adaptor molecule (STAM), mitogen-activated protein kinase (MAPK) and the interacting proteins.

**Table 6:** Putative immune genes involved in JAK-STAT pathway.

Signaling molecular	Unigene	ID	E-value	Description
**TPO**	comp107905_c0	XP_002431634.1	0	Thyroid peroxidase precursor, putative [*Pediculus humanus corporis*]
	comp13197_c0	XP_002431634.1	0	Thyroid peroxidase precursor, putative [*Pediculus humanus corporis*]
	comp13197_c1	XP_002431634.1	0	Thyroid peroxidase precursor, putative [*Pediculus humanus corporis*]
	comp170056_c0	XP_002431634.1	0	Thyroid peroxidase precursor, putative [*Pediculus humanus corporis*]
	comp267202_c0	XP_002431634.1	0	Thyroid peroxidase precursor, putative [*Pediculus humanus corporis*]
**CytokinR**	comp14984_c0	EFN76806.1	1.02E-75	Cytokine receptor [*Harpegnathos saltator*]
	comp23666_c0	EFN76806.1	1.02E-75	Cytokine receptor [*Harpegnathos saltator*]
	comp23666_c1	EFN76806.1	1.02E-75	Cytokine receptor [*Harpegnathos saltator*]
	comp97600_c0	EEZ97840.1	5.51E-22	hypothetical protein TcasGA2_TC000209 [*Tribolium castaneum*]
	comp155435_c0	ADV57398.1	3.34E-69	leptin receptor protein [*Eriocheir sinensis*]
	comp30992_c0	ADV57398.1	3.34E-69	leptin receptor protein [*Eriocheir sinensis*]
**JAK**	comp11170_c0	XP_002425471.1	3.44E-49	tyrosine-protein kinase jak2, putative [*Pediculus humanus corporis*]
	comp13215_c0	XP_002425471.1	3.44E-49	tyrosine-protein kinase jak2, putative [*Pediculus humanus corporis*]
	comp29254_c0	XP_002425471.1	5.67E-122	tyrosine-protein kinase jak2, putative [*Pediculus humanus corporis*]
	comp29596_c1	XP_002425471.1	5.67E-122	tyrosine-protein kinase jak2, putative [*Pediculus humanus corporis*]
**STAT**	comp25948_c0	ACA79939.1	0	STAT long form [*Penaeus monodon*]
**STAM**	comp28940_c0	XP_003398833.1	1.29E-104	PREDICTED: signal transducing adapter molecule 1-like [*Bombus terrestris*]
**CBL**	comp10185_c0	XP_002428625.1	0	E3 ubiquitin-protein ligase CBL, putative [*Pediculus humanus corporis*]
	comp10185_c1	XP_002428625.1	0	E3 ubiquitin-protein ligase CBL, putative [*Pediculus humanus corporis*]
**PIAS**	comp22323_c0	EFN82639.1	5.21E-134	E3 SUMO-protein ligase PIAS2 [*Harpegnathos saltator*]
**CBP**	comp25273_c0	XP_002423797.1	1.26E-88	CREB-binding protein, putative [*Pediculus humanus corporis*]
	comp30717_c0	EFN64132.1	6.83E-111	CREB-binding protein [*Camponotus floridanus*]
**SOCS**	comp24182_c1	EGI69666.1	2.54E-85	Suppressor of cytokine signaling 5 [*Acromyrmex echinatior*]
	comp12564_c0	XP_001603336.1	8.47E-94	PREDICTED: similar to CG8146-PA [*Nasonia vitripennis*]
	comp12564_c1	XP_001603336.1	8.47E-94	PREDICTED: similar to CG8146-PA [*Nasonia vitripennis*]
	comp13447_c0	XP_001603336.1	8.47E-94	PREDICTED: similar to CG8146-PA [*Nasonia vitripennis*]
	comp73922_c0	XP_001603336.1	8.47E-94	PREDICTED: similar to CG8146-PA [*Nasonia vitripennis*]
**Pim**	comp126988_c0	NP_001090165.1	1.67E-89	pim-3 oncogene [*Xenopus laevis*]
	comp188941_c0	NP_001090165.1	1.67E-89	pim-3 oncogene [*Xenopus laevis*]
**SHP2**	comp24266_c1	XP_002430772.1	2.00E-18	tyrosine-protein phosphatase corkscrew, putative [*Pediculus humanus corporis*]
**GRB2**	comp30475_c0	XP_969998.1	2.03E-105	PREDICTED: similar to AGAP011768-PA [*Tribolium castaneum*]
	comp30475_c1	XP_969998.1	2.03E-105	PREDICTED: similar to AGAP011768-PA [*Tribolium castaneum*]
**SOS**	comp27781_c0	XP_002428152.1	0	ras GTP exchange factor, son of sevenless, putative [*Pediculus humanus corporis*]
	comp30785_c2	XP_002428152.1	0	ras GTP exchange factor, son of sevenless, putative [*Pediculus humanus corporis*]
**PI3K**	comp31023_c0	ADE44091.1	0	phosphoinositide 3-kinase isoform b [*Panulirus argus*]
	comp24583_c0	XP_001606345.1	0	PREDICTED: similar to MGC80357 protein [*Nasonia vitripennis*]
**AKT**	comp29921_c0	ADM87425.3	0	Akt [Gecarcinus lateralis]
**CycD**	comp12912_c0	NP_001089817.1	1.95E-55	cyclin D2 [*Xenopus laevis*]
	comp138262_c0	NP_001089817.1	1.95E-55	cyclin D2 [*Xenopus laevis*]
	comp28643_c0	XP_974376.1	5.44E-50	PREDICTED: similar to cyclin d [*Tribolium castaneum*]
**Myc**	comp28588_c2	EFX79343.1	9.02E-16	Myc, dMyc-like protein [*Daphnia pulex*]
	comp30540_c3	EEZ99541.1	2.98E-15	hypothetical protein TcasGA2_TC000123 [*Tribolium castaneum*]
**BclXL**	comp27005_c0	EGI69168.1	2.27E-20	Bcl-2-like protein 1 [*Acromyrmex echinatior*]
**Spred**	comp25472_c2	XP_002414876.1	3.24E-41	sprouty protein evh1 domain-containing protein, putative [*Ixodes scapularis*]
	comp28599_c0	XP_002414876.1	3.24E-41	sprouty protein evh1 domain-containing protein, putative [*Ixodes scapularis*]
	comp28599_c4	NP_001164144.1	1.05E-46	sprouty-related protein with EVH-1 domain [*Tribolium castaneum*]
**Sprouty**	comp22483_c0	EFZ18471.1	4.90E-45	hypothetical protein SINV_11790 [*Solenopsis invicta*]

#### MAPK pathway

Putative MAPK signaling pathway containing 122 non-redundant unigenes was also analyzed (Table 7, Figure S4). MAPKs were composed of three different major families – c-Jun N-terminal kinase (JNK) family, p38/stress-activated protein kinase (p38/SAPK) family and extracellular-signal regulated kinase (ERK) family, and regulated different processes by protease cascade. In this putative pathway, each cascade was triggered by extracellular signals and resulted in activation of MAPK kinase kinase (MAPKKK/MEKK), followed by activation of MAPK kinase (MAPKK/MEK/MKK) and MAPK/ERK, finally leading to function of diverse substrates and NF-kB proteins.

**Table 7:** Putative immune genes involved in MAPK pathway.

Signaling molecular	Unigene	ID	E-value	Description
**CACN**	comp371_c0	XP_003251102.1	0	PREDICTED: voltage-dependent calcium channel subunit alpha-2/delta-3-like [*Apis mellifera*]
	comp21877_c0	XP_003251102.1	0	PREDICTED: voltage-dependent calcium channel subunit alpha-2/delta-3-like [*Apis mellifera*]
	comp232994_c0	XP_003251102.1	0	PREDICTED: voltage-dependent calcium channel subunit alpha-2/delta-3-like [*Apis mellifera*]
	comp398239_c0	XP_003251102.1	0	PREDICTED: voltage-dependent calcium channel subunit alpha-2/delta-3-like [*Apis mellifera*]
	comp10818_c0	XP_002168351.1	3.42E-15	PREDICTED: similar to calcium channel, voltage-dependent, alpha2/delta subunit 1, partial [*Hydra magnipapillata*]
	comp241026_c0	XP_002742061.1	1.68E-62	PREDICTED: calcium channel, voltage-dependent, alpha2/delta subunit 3-like [*Saccoglossus kowalevskii*]
	comp409663_c0	XP_001807530.1	8.88E-45	PREDICTED: similar to voltage-gated calcium channel alpha 1 subunit [*Tribolium castaneum*]
**EGFR**	comp15094_c1	XP_003395927.1	0	PREDICTED: epidermal growth factor receptor-like [*Bombus terrestris*]
	comp19914_c0	XP_003395927.1	0	PREDICTED: epidermal growth factor receptor-like [*Bombus terrestris*]
	comp108074_c0	XP_003395927.1	0	PREDICTED: epidermal growth factor receptor-like [*Bombus terrestris*]
	comp190699_c0	XP_003395927.1	0	PREDICTED: epidermal growth factor receptor-like [*Bombus terrestris*]
**FGFR**	comp3098_c0	XP_003401483.1	0	PREDICTED: fibroblast growth factor receptor homolog 1-like [*Bombus terrestris*]
	comp15223_c0	XP_003401483.1	0	PREDICTED: fibroblast growth factor receptor homolog 1-like [*Bombus terrestris*]
	comp127712_c0	XP_003401483.1	0	PREDICTED: fibroblast growth factor receptor homolog 1-like [*Bombus terrestris*]
	comp143525_c0	XP_003401483.1	0	PREDICTED: fibroblast growth factor receptor homolog 1-like [*Bombus terrestris*]
	comp226442_c0	XP_003401483.1	0	PREDICTED: fibroblast growth factor receptor homolog 1-like [*Bombus terrestris*]
	comp290904_c0	XP_003401483.1	0	PREDICTED: fibroblast growth factor receptor homolog 1-like [*Bombus terrestris*]
	comp20492_c0	CAH03726.1	1.41E-49	TPA: FGF receptor-like protein 1a [*Takifugu rubripes*]
	comp25479_c0	CAH03726.1	1.41E-49	TPA: FGF receptor-like protein 1a [*Takifugu rubripes*]
	comp127203_c0	CAH03726.1	1.41E-49	TPA: FGF receptor-like protein 1a [*Takifugu rubripes*]
	comp22034_c0	NP_001012263.2	1.30E-22	fibroblast growth factor receptor-like 1b [*Danio rerio*]
**PDGFR**	comp4550_c0	XP_002422693.1	4.28E-19	alpha platelet-derived growth factor receptor precursor, putative [*Pediculus humanus corporis*]
**GRB2**	comp30475_c0	XP_969998.1	2.03E-105	PREDICTED: similar to AGAP011768-PA [*Tribolium castaneum*]
**SOS**	comp27781_c0	XP_002428152.1	0	ras GTP exchange factor, son of sevenless, putative [*Pediculus humanus corporis*]
	comp30785_c2	XP_002428152.1	0	ras GTP exchange factor, son of sevenless, putative [*Pediculus humanus corporis*]
**Ras**	comp23549_c0	XP_972376.2	1.94E-37	PREDICTED: similar to MRAS2, putative [*Tribolium castaneum*]
	comp24659_c0	XP_972154.1	3.89E-20	PREDICTED: similar to MRAS2, putative [*Tribolium castaneum*]
	comp27508_c0	AAK14389.1	1.34E-89	Ras [*Marsupenaeus japonicus*]
	comp162483_c0	XP_393895.2	1.14E-78	PREDICTED: ras-related protein M-Ras-like [*Apis mellifera*]
	comp29718_c0	XM_003506401.1	3.01E-14	PREDICTED: *Cricetulus griseus* ras-related protein R-Ras2-like [*Cricetulus griseus*]
**G12**	comp16378_c0	EFX86199.1	8.35E-18	guanine nucleotide binding protein, gamma subunit [*Daphnia pulex*]
	comp18258_c0	EFX86199.1	8.35E-18	guanine nucleotide binding protein, gamma subunit [*Daphnia pulex*]
	comp26378_c0	EGI64184.1	3.12E-133	Guanine nucleotide-binding protein subunit alpha-like protein [*Acromyrmex echinatior*]
**Gap1m**	comp24182_c3	XP_001945701.2	0	PREDICTED: probable Ras GTPase-activating protein-like [*Acyrthosiphon pisum*]
	comp193916_c0	XP_001945701.2	2.60E-111	PREDICTED: probable Ras GTPase-activating protein-like [*Acyrthosiphon pisum*]
**p120GAP**	comp30738_c0	XP_001942745.1	0	PREDICTED: ras GTPase-activating protein 1-like [*Acyrthosiphon pisum*]
**NF1**	comp16823_c0	XP_003402236.1	3.29E-64	PREDICTED: neurofibromin-like [*Bombus terrestris*]
	comp29735_c1	XP_001602698.1	0	PREDICTED: similar to neurofibromin [*Nasonia vitripennis*]
**CNrasGEF**	comp21203_c0	XP_001952587.1	0	PREDICTED: rap guanine nucleotide exchange factor 2-like [*Acyrthosiphon pisum*]
	comp2527_c0	XP_002732773.1	3.29E-28	PREDICTED: Rap guanine nucleotide exchange factor 2-like [*Saccoglossus kowalevskii*]
	comp398736_c0	XP_002732773.1	3.29E-28	PREDICTED: Rap guanine nucleotide exchange factor 2-like [*Saccoglossus kowalevskii*]
**PKA**	comp11911_c1	XP_002423550.1	0	cAMP-dependent protein kinase catalytic subunit, putative [*Pediculus humanus corporis*]
	comp14993_c0	XP_002423550.1	0	cAMP-dependent protein kinase catalytic subunit, putative [*Pediculus humanus corporis*]
	comp29154_c0	XP_973065.1	3.14E-133	PREDICTED: similar to camp-dependent protein kinase catalytic subunit [*Tribolium castaneum*]
**PKC**	comp4496_c0	XP_001601074.1	1.45E-34	PREDICTED: similar to conventional protein kinase C [*Nasonia vitripennis*]
	comp15449_c0	XP_001601074.1	0	PREDICTED: similar to conventional protein kinase C [*Nasonia vitripennis*]
	comp99841_c0	XP_001601074.1	0	PREDICTED: similar to conventional protein kinase C [*Nasonia vitripennis*]
	comp15879_c1	XP_002410223.1	5.10E-48	protein kinase C, putative [*Ixodes scapularis*]
	comp159920_c0	XP_002410223.1	5.10E-48	protein kinase C, putative [*Ixodes scapularis*]
	comp225802_c0	XP_002410223.1	5.10E-48	protein kinase C, putative [*Ixodes scapularis*]
**Rap1**	comp29806_c0	ACJ66625.1	7.77E-90	Ras protein [*Fenneropenaeus chinensis*]
**IKK**	comp17756_c0	AAX56336.1	3.37E-90	ikk-like protein [*Pinctada fucata*]
	comp27376_c0	AAC05683.1	5.60E-56	I-kappa-B kinase [*Crassostrea gigas*]
**NF-kB**	comp27894_c0	ADM14334.1	1.03E-168	relish [*Eriocheir sinensis*]
	comp29150_c7	ADM14334.1	0	relish [*Eriocheir sinensis*]
**ERK**	comp19175_c0	NP_001036922.1	1.90E-158	MAP kinse-ERK kinase [*Bombyx mori*]
	comp26529_c0	NP_001036922.1	0	mitogen-activated protein kinase [*Scylla paramamosain*]
**Tau**	comp23830_c0	XP_001955318.1	1.60E-56	GF18699 [*Drosophila ananassae*]
**STMN**	comp412415_c0	EGI59233.1	8.12E-49	Stathmin-4 [*Acromyrmex echinatior*]
**cPLA2**	comp31173_c1	XP_002127884.1	9.58E-53	PREDICTED: similar to Cytosolic phospholipase A2 (cPLA2) (Phospholipase A2 group IVA) [*Ciona intestinalis*]
**MNK1/2**	comp21112_c0	ACY66411.1	2.83E-167	map kinase-interacting serine/threonine [*Scylla paramamosain*]
**RSK2**	comp29304_c1	XP_002432758.1	9.42E-24	Ribosomal protein S6 kinase alpha-2, putative [*Pediculus humanus corporis*]
**Elk-1**	comp26462_c0	XP_002429096.1	8.37e-67	protein C-ets-1-B, putative [*Pediculus humanus corporis*]
**Sapla**	comp30605_c0	XP_002410379.1	2.39E-47	ETS domain-containing protein Elk-4, putative [*Ixodes scapularis*]
**Myc**	comp24902_c0	EFN80642.1	3.61E-17	C-myc promoter-binding protein [*Harpegnathos saltator*]
	comp28588_c2	EFX79343.1	9.02E-16	Myc, dMyc-like protein [*Daphnia pulex*]
**SRF**	comp30221_c2	CAB62047.1	5.35E-51	Serum Response Factor [*Artemia franciscana*]
**MKP**	comp25626_c0	XP_002430571.1	4.24E-115	dual specificity protein phosphatase, putative [*Pediculus humanus corporis*]
**PPP3C**	comp5822_c0	XM_001369081.1	0	PREDICTED: similar to calcineurin A [*Nasonia vitripennis*]
	comp20629_c3	XP_001602102.1	0	PREDICTED: similar to calcineurin A [*Nasonia vitripennis*]
	comp110375_c0	XP_001602102.1	0	PREDICTED: similar to calcineurin A [*Nasonia vitripennis*]
	comp30630_c4	ADD19580.1	1.35E-85	Ca2+/calmodulin-dependent protein phosphatase [*Glossina morsitans morsitans*]
**FASL**	comp16267_c0	AEK86525.1	3.86E-80	TNFSF [*Litopenaeus vannamei*]
	comp30406_c0	AEK86525.1	3.86E-80	TNFSF [*Litopenaeus vannamei*]
**FAS**	comp18918_c0	AEK86527.1	8.05E-34	TNFRSF [*Litopenaeus vannamei*]
	comp169288_c0	AEK86527.1	8.05E-34	TNFRSF [*Litopenaeus vannamei*]
	comp160283_c0	AEK86527.1	8.05E-34	TNFRSF [*Litopenaeus vannamei*]
**TGFBR**	comp18124_c0	XP_002412676.1	2.15E-160	transforming growth factor-beta receptor type I, putative [*Ixodes scapularis*]
**CASP**	comp10698_c0	ADM45311.1	3.38E-27	caspase [*Eriocheir sinensis*]
	comp28182_c0	ADM45311.1	6.08E-32	caspase [*Eriocheir sinensis*]
	comp16422_c0	XP_003385047.1	1.33E-20	PREDICTED: caspase-3-like [*Amphimedon queenslandica*]
	comp165956_c0	XP_003385047.1	1.33E-20	PREDICTED: caspase-3-like [*Amphimedon queenslandica*]
	comp419824_c0	XP_003385047.1	1.33E-20	PREDICTED: caspase-3-like [*Amphimedon queenslandica*]
**DAXX**	comp22998_c0	XP_002735579.1	9.16E-29	PREDICTED: death-domain associated protein-like [*Saccoglossus kowalevskii*]
**ECSIT**	comp22998_c0	BAI40012.1	4.42E-114	evolutionarily conserved signaling intermediate in Toll pathways [*Marsupenaeus japonicus*]
**PP2CB**	comp28743_c0	NP_001008030.1	1.21E-134	protein phosphatase, Mg2+/Mn2+ dependent, 1B [*Xenopus (Silurana) tropicalis*]
**cdc42/Rac**	comp22273_c2	XP_002428346.1	3.00E-98	RAC GTPase, putative [*Pediculus humanus corporis*]
	comp86099_c0	XP_002428346.1	3.00E-98	RAC GTPase, putative [*Pediculus humanus corporis*]
	comp29231_c2	XP_001660307.1	1.27E-98	rac gtpase [*Aedes aegypti*]
**HGK**	comp23927_c1	XP_003403321.1	1.79E-16	PREDICTED: mitogen-activated protein kinase kinase kinase kinase 4-like isoform 1 [*Bombus terrestris*]
**PAK1/2**	comp14636_c1	EGI64863.1	6.98E-114	Serine/threonine-protein kinase PAK 1 [*Acromyrmex echinatior*]
	comp25614_c0	EGI64863.1	6.98E-114	Serine/threonine-protein kinase PAK 1 [*Acromyrmex echinatior*]
	comp26148_c0	XP_003251334.1	0	PREDICTED: serine/threonine-protein kinase PAK 1 isoform 2 [*Apis mellifera*]
	comp26487_c0	XP_002426989.1	1.33E-139	CDC42 GTPase-activating protein, putative [*Pediculus humanus corporis*]
**MST1/2**	comp28042_c0	EGI57844.1	1.02E-157	Serine/threonine-protein kinase 3 [*Acromyrmex echinatior*]
	comp98224_c0	EGI57844.1	1.02E-157	Serine/threonine-protein kinase 3 [*Acromyrmex echinatior*]
**MEKK1**	comp27862_c0	XP_002408296.1	1.02E-21	mitogen activated protein kinase kinase kinase 1, MAPKKK1, MEKK1, putative [*Ixodes scapularis*]
	comp29482_c2	XP_424734.2	1.27E-44	PREDICTED: similar to MEK kinase 1 [*Gallus gallus*]
	comp64508_c0	XP_424734.2	1.27E-44	PREDICTED: similar to MEK kinase 1 [*Gallus gallus*]
**LZK**	comp26893_c0	XP_003396640.1	1.42E-164	PREDICTED: mitogen-activated protein kinase kinase kinase 13-like isoform 2 [*Bombus terrestris*]
**TAK1**	comp10992_c0	EFN79813.1	1.13E-92	Mitogen-activated protein kinase kinase kinase 7 [*Harpegnathos saltator*]
**MEKK4**	comp81382_c0	EDL02074.1	0	mCG16678 [Mus musculus]
**TAO**	comp28070_c0	XP_002426013.1	0	predicted protein [Pediculus humanus corporis]
**FLNA**	comp27356_c0	EFX70014.1	2.10E-12	hypothetical protein DAPPUDRAFT_328543 [*Daphnia pulex*]
	comp31197_c0	XP_002423351.1	1.41E-35	Filamin-C, putative [*Pediculus humanus corporis*]
**JIP3**	comp6235_c0	XM_003354659.1	5.17E-37	PREDICTED: Sus scrofa mitogen-activated protein kinase 8 interacting protein 3, transcript variant 2 (MAPK8IP3) [*Sus scrofa*]
	comp30482_c0	XP_003395970.1	0	PREDICTED: LOW QUALITY PROTEIN: JNK-interacting protein 3-like [*Bombus terrestris*]
**HSP72**	comp8227_c0	XP_002649823.1	4.32E-80	molecular chaperone [*Enterocytozoon bieneusi H348*]
	comp133984_c0	AAS57912.1	4.91E-65	70 kDa heat shock cognate protein 1 [*Vigna radiata*]
	comp197937_c0	ACB70177.1	2.10E-49	70 kDa heat shock protein [*Capparis spinosa*]
	comp406329_c0	XP_002532297.1	1.19E-173	heat shock protein, putative [*Ricinus communis*]
	comp25846_c0	ACF98297.1	0	heat shock protein 70 [*Eriocheir sinensis*]
**ARRB**	comp28931_c4	XM_001867259.1	8.49E-26	*Culex quinquefasciatus* beta-arrestin 1
**Crk**	comp28991_c0	XP_002427598.1	5.89E-106	Adapter molecule Crk, putative [*Pediculus humanus corporis*]
**MKK4**	comp25769_c0	EFN81517.1	4.92E-152	Dual specificity mitogen-activated protein kinase kinase 4 [*Harpegnathos saltator*]
**JNK**	comp20212_c0	BAI87826.1	0	c-jun N-terminal kinase [*Marsupenaeus japonicus*]
**JUN**	comp28673_c0	EGI68820.1	1.90E-49	Transcription factor AP-1 [*Acromyrmex echinatior*]
	comp31162_c1	EGI68820.1	1.90E-49	Transcription factor AP-1 [*Acromyrmex echinatior*]
**AKT**	comp29921_c0	ADM87425.3	0	Akt [*Gecarcinus lateralis*]
**PP5**	comp29577_c0	XP_971407.1	0	PREDICTED: similar to protein phosphatase-5 [*Tribolium castaneum*]
**ATF2**	comp30056_c0	XP_001515843.1	1.13E-16	PREDICTED: similar to activating transcription factor 2 [*Ornithorhynchus anatinus*]
**p38**	comp24454_c0	ADT91683.1	8.07E-169	p38 mitogen-activated protein kinase [*Apis cerana cerana*]
**p53**	comp24379_c1	ACQ58385.1	2.54E-13	p53 and DNA damage-regulated protein 1 [*Anoplopoma fimbria*]
	comp204793_c0	XP_968601.2	2.59E-102	PREDICTED: similar to apoptosis stimulating of P53 [*Tribolium castaneum*]
**MAX**	comp25261_c3	XP_003401810.1	7.59E-38	PREDICTED: protein max-like isoform 1 [*Bombus terrestris*]
**MEF2C**	comp18140_c0	XP_971771.1	1.56E-82	PREDICTED: similar to myocyte-specific enhancer factor 2d [*Tribolium castaneum*]
	comp18140_c1	XP_971771.1	1.56E-82	PREDICTED: similar to myocyte-specific enhancer factor 2d [*Tribolium castaneum*]
**MSK1/2**	comp21333_c0	XP_002431024.1	3.20E-21	Ribosomal protein S6 kinase alpha-5, putative [*Pediculus humanus corporis*]
**NLK**	comp17966_c0	XP_002048311.1	0	GJ13897 [Drosophila virilis]
**MAPKAPK**	comp15249_c0	ABC25082.1	7.31E-87	MAP kinase activated protein-kinase-2 [*Glossina morsitans morsitans*]
	comp30931_c0	ABC25082.1	7.31E-87	MAP kinase activated protein-kinase-2 [*Glossina morsitans morsitans*]
	comp144964_c0	ABC25082.1	7.31E-87	MAP kinase activated protein-kinase-2 [*Glossina morsitans morsitans*]

### Validation of Illumina sequencing results by RT-PCR

Quantitative RT-PCR was used to confirm the expression profiles of genes that were identified in Illumina sequencing analysis. As was shown in Table S1, analyzed members of the study contained some pathway-associated components, including IMD, SOCS, Spatzle (Spa), filamin (FLNA), p21-activated kinase 1 (Pak1), cytosolic phospholipase A2 (cPLA2), dual-specificity MAP kinase phosphatase (MKP), heat shock 70kDa protein (HSP), Ras-related C3 botulinum toxin substrate 1 (Rac1), hepatocyte growth factor (HGF), vascular endothelial growth factor receptor (VEGFR). Many other important immune-related genes, such as arginase (ARG), chitinase (Chi), Integrin (Int), lysozyme (LZM), peroxinectin (Pero), thymosin (Thy), anti-lipopolysaccharide factor (ALF), mannose receptor (ManR), scavenger-receptor (ScaR), mannose-binding protein (MBL), masquerade-like protein (MasL), glutathione peroxidase (GPX), glutathione S-transferase (GST), thioredoxin reductase (TrxR), trypsin-like serine protease (TrySP), chymotrypsin-like serine protease (ChySP), were also identified and analyzed (Table S1).

**Figure 6:**
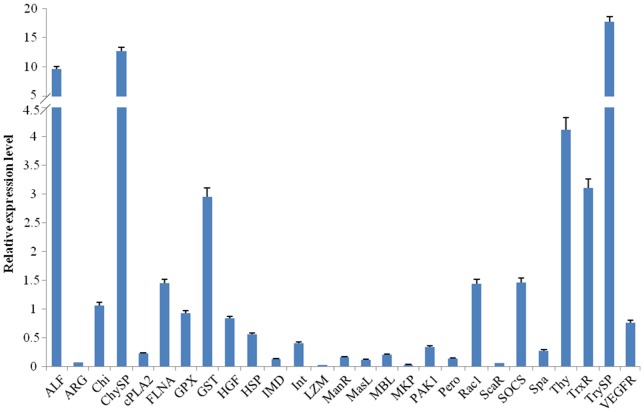
Real-time PCR validation of the expressed genes in Illumina sequencing.

Results of RT-PCR revealed different expression abundances of the analyzed genes (Figure 6). Among them, TrySP, ChySP and ALF showed highest expression level, followed by Thy and TrxR, while LZW displayed the lowest level. It was consistent with the results of Illumina sequencing data (Table S1), which not only validated the expression profile of different identified immune genes, but also verified the reliability and accuracy of our transcriptome analysis.

## Discussion

In this context, considerable efforts have been made to research hepatopancreas transcriptome of microbial challenged *E. sinensis* by high-throughput sequencing technology (Solexa/Illumina). Comparing with EST analysis of hepatopancreas from *E. sinensis* with traditional method [19], [22], our study produces more sequencing reads and assembled unigenes. It largely enriches transcriptome data of *E. sinensis* and indicates enormous advantage of high-throughput technology. Although a comparative transcriptome analysis of haemocytes from *E. sinensis* under normal condition and in response to *Spiroplasma eriocheiris* infection indicates certain microRNAs may be essential in interaction between host and pathogen [11], only miRNAs are identified and analyzed for the expression pattern. In our study, various immune genes and pathways are annotated from hepatopancreas of *E. sinensis* after immune challenge. The analysis increases molecular information and genomic resources of *E. sinensis* in response to microorganism stimulation.

Toll pathway was initially identified in genetic screen of genes involved in early embryonic development of *Drosophila* [23] and gradually studied of importance in innate immunity. In economic crustaceans, many genes related to Toll pathway, such as Spatzle [24], Toll [24], MyD88 [25], Pelle [26] and TRAF6 [27], have been reported from shrimp, while only SpToll of *Scylla paramamosain* [28] has been cloned and characterized from crab. In the present study, we are first to find various key members of Toll pathway in *E. sinensis*. This suggests the existence of putative Toll pathway in crab and indicates its crucial function in antimicrobial response. Moreover, different from mammalian Toll-like receptors (TLRs) directly functioning as a pattern recognition receptor (PRR) to recognize pathogen-associated molecular patterns (PAMPs) [29], DmToll of *Drosophila melanogaster* uses the cytokine-like molecule Spatzle as a ligand [24], [30], [31]. In *E. sinensis*, identification of Spatzle in our study also suggests that the Chinese mitten crab Toll may be activated by functioning with Spatzle. In addition, in spite of different MyD88 variants in human, mice, chicken and other vertebrates, only a MyD88 variant gene is found in an invertebrate species *L. vannamei* [25]. Here, we find one MyD88 sequence of *E. sinensis* that shows highest similarility to homolog from *L. vannamei*. This will provide a foundation for further study of MyD88 in crab.

Gram-negative bacteria-yielded diaminopimelic acid (DAP)-type peptidoglycan can be recognized by peptidoglycan recognition protein (PGRP)-LE and PGRP-LC receptor complex, which then activate IMD and cause activation of signaling cascade to trigger Relish [32]. Experiments of *Drosophila* also reveal that infection by Gram-negative bacteria activates IMD pathway, but not Toll pathway [30], [31]. In this context, among different molecules relevant to IMD pathway, not only caspase and Relish previously reported [33], [34] are identified, but also IMD, dTAK1 and IKK are first found in microbial challenged *E. sinensis*. Similarly, LvIMD of *L. vannamei* and FcRelish of *Fenneropenaeus chinensis* are identified after immune challenge and characterization of them implies that they can induce expression of some antimicrobial peptides (AMPs), which are integral components of innate immune system and exhibit great activities to defense against pathogens [35], [36]. Taken these reports together, investigation of principal component molecules will promote researching on innate immune mechanism and immune pathway of *E. sinensis*.

A large number of molecules involved in JAK-STAT signaling pathway such as four JAKs, seven STATs and more than 30 cytokines are widely found in mammals [21]. However, only SOCS and leptin receptor protein (LEPR) have been cloned from *E. sinensis* [37], [38]. In the present study, along with SOCS and LEPR, many other genes including CytokineR, JAK, STAT, downstream genes and regulatory molecules (CIS, SHP1, SOCS, PIAS and STAM) are first fully and systemically identified in crab. Considering different aspects of cell development and host response activated by JAK-STAT pathway [39], there is no surprise that lots of regulators are found to control this pathway. Expression and regulation of components in JAK-STAT pathway are also reported in transcriptome analyses of microbial infected *Pseudosciaena crocea* [40] and *Laodelphax striatellus* [41]. These reports together increase knowledge of JAK-STAT pathway on microbial stimulation and provide valuable information for further study of immune response against pathogen infection. Additionally, researchers have compared the one single STAT gene from invertebrates with seven STATs from vertebrates by phylogenetic analysis [42]. This comparison supports the hypothesis that STAT genes duplicate before splitting in invertebrates and vertebrates and shows difference between them. In mammals and other vertebrates, JAK-STAT pathway plays a crucial function in lots of biological processes of both innate and adaptive immunity, such as apoptosis, proliferation, differentiation, hematopoiesis, oncogenesis and immune defense [39], [43]. However, in crustaceans, only the antibacterial or antiviral activities of several JAK/STAT genes are known so far [37], [44], [45]. It is still unknown whether the pathway has other functions and needs us to do more efforts for its complete function research.

MAPK pathway widely exists in all eukaryotes from yeast to human. Through a conserved three-kinase cascade that finally phosphorylates intracellular substrates and transcription factors, it transducts extracellular cues to cytoplasm and nucleus to control physiological processes [46]. Currently, similar with JAK-STAT pathway, most knowledge of MAPK pathway is also focused on vertebrate system. In vertebrates, this pathway is multifunctional and plays a key role in anti-stress, reproduction, cell development, differentiation and inflammation [46]. In shrimp, anti-lipopolysaccharide factor treatment can regulate *Trichomonas vaginalis*-induced proinflammatory cytokines through MAPK pathway [47]. Interestingly, in the crab *Chasmagnathus*, MAPK pathway participates in neural plasticity, which can only be found in rodents and mollusks before, and is necessary for long-term memory consolidation of this crab model [48]. Thus, MAPK pathway may have many different functions in various species of vertebrates and crustaceans. However, for the reason that knowledge about MAPK pathway in aquatic invertebrates is largely unclear, it still needs deep research to fully clarify the role of this pathway. Our detection of numerous genes involved in MAPK pathway, such as ERK, JNK, p38, MEK, MEKK, Elk, Crk and CREB, will offer valuable reference in crab and other important crustaceans.

In conclusion, numerous genes from hepatopancreas of microbial challenged *E. sinensis* are characterized to be associated with Toll, IMD, JAK-STAT and MAPK pathways. Accuracy of Illumina sequencing data and expression profile of the identified genes are also further confirmed by RT-PCR. This research will be not only helpful to fully research host-pathogen interaction and comprehensively understand immune system of crab, but also beneficial to prevent diseases appeared in crab culture.

## Supporting Information

Figure S1 **Putative Toll pathway.** Putative Toll pathway of *E. sinensis* was constructed based on knowledge in *Drosophila* and shrimps. Proteins appearing in hepatopancreas of microbial challenged *E. sinensis* were represented in grey circle and absent proteins in grey square. However, most interactions have to be confirmed experimentally.
(TIF)

Figure S2 **Putative IMD pathway.** Putative IMD pathway of *E. sinensis* was constructed based on knowledge in *Drosophila* and shrimps. Proteins appearing in hepatopancreas of microbial challenged *E. sinensis* were represented in grey circle and absent proteins in grey square. However, most interactions have to be confirmed experimentally.
(TIF)

Figure S3 **Putative JAK-STAT pathway.** Putative JAK-STAT pathway of *E. sinensis* was constructed based on KEGG reference pathway. Proteins appearing in hepatopancreas of microbial challenged *E. sinensis* were represented in circle and absent proteins in square. However, most interactions have to be confirmed experimentally.
(TIF)

Figure S4 **Putative MAPK pathway.** Putative MAPK pathway of *E. sinensis* was constructed based on KEGG reference pathway. Proteins appearing in hepatopancreas of microbial challenged *E. sinensis* were represented in grey circle and absent proteins in grey square. However, most interactions have to be confirmed experimentally.
(TIF)

Table S1 **Genes and specific primers used for real-time PCR.**
(DOC)
