# Supplementary figures and images for: Transcriptome Analysis and Discovery of Genes Involved in Immune Pathways from Hepatopancreas of Microbial Challenged Mitten Crab Eriocheir sinensis
Source: PLoS One. 2013 Jul 17;8(7):e68233. doi: 10.1371/journal.pone.0068233 (PMC3714283; doi:10.1371/journal.pone.0068233)

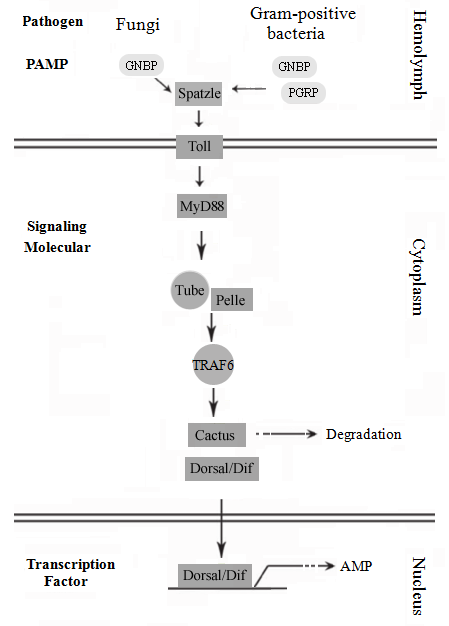

Supplement: Figure S1 — Putative Toll pathway. Putative Toll pathway of E. sinensis was constructed based on knowledge in Drosophila and shrimps. Proteins appearing in hepatopancreas of microbial challenged E. sinensis were represented in grey circle and absent proteins in grey square. However, most interactions have to be confirmed experimentally. (TIF) [file pone.0068233.s001.tif]

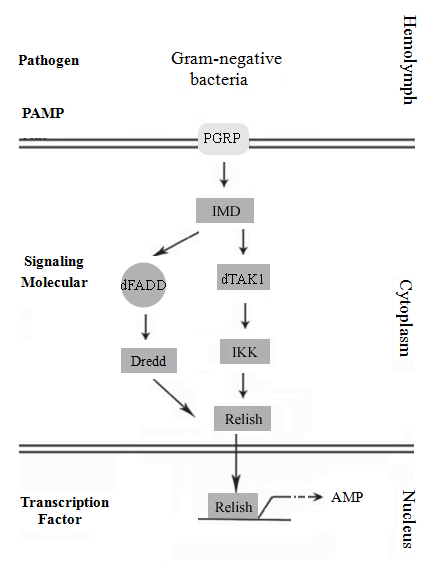

Supplement: Figure S2 — Putative IMD pathway. Putative IMD pathway of E. sinensis was constructed based on knowledge in Drosophila and shrimps. Proteins appearing in hepatopancreas of microbial challenged E. sinensis were represented in grey circle and absent proteins in grey square. However, most interactions have to be confirmed experimentally. (TIF) [file pone.0068233.s002.tif]

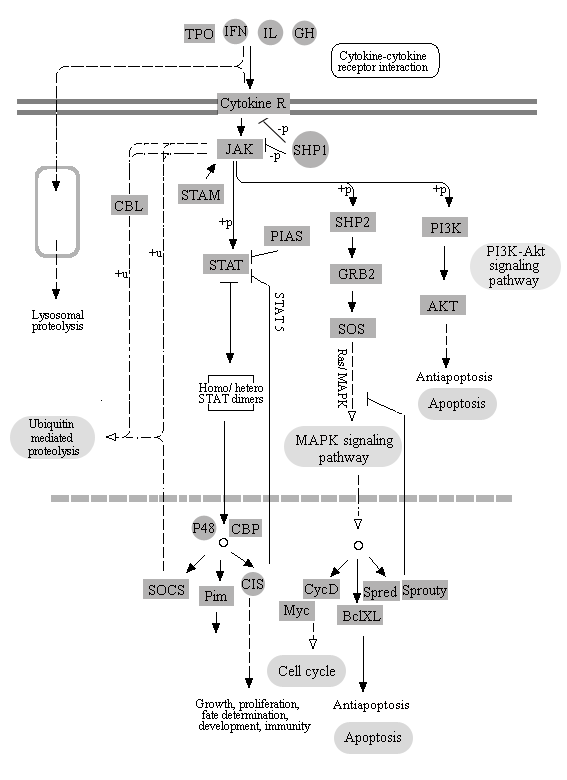

Supplement: Figure S3 — Putative JAK-STAT pathway. Putative JAK-STAT pathway of E. sinensis was constructed based on KEGG reference pathway. Proteins appearing in hepatopancreas of microbial challenged E. sinensis were represented in circle and absent proteins in square. However, most interactions have to be confirmed experimentally. (TIF) [file pone.0068233.s003.tif]

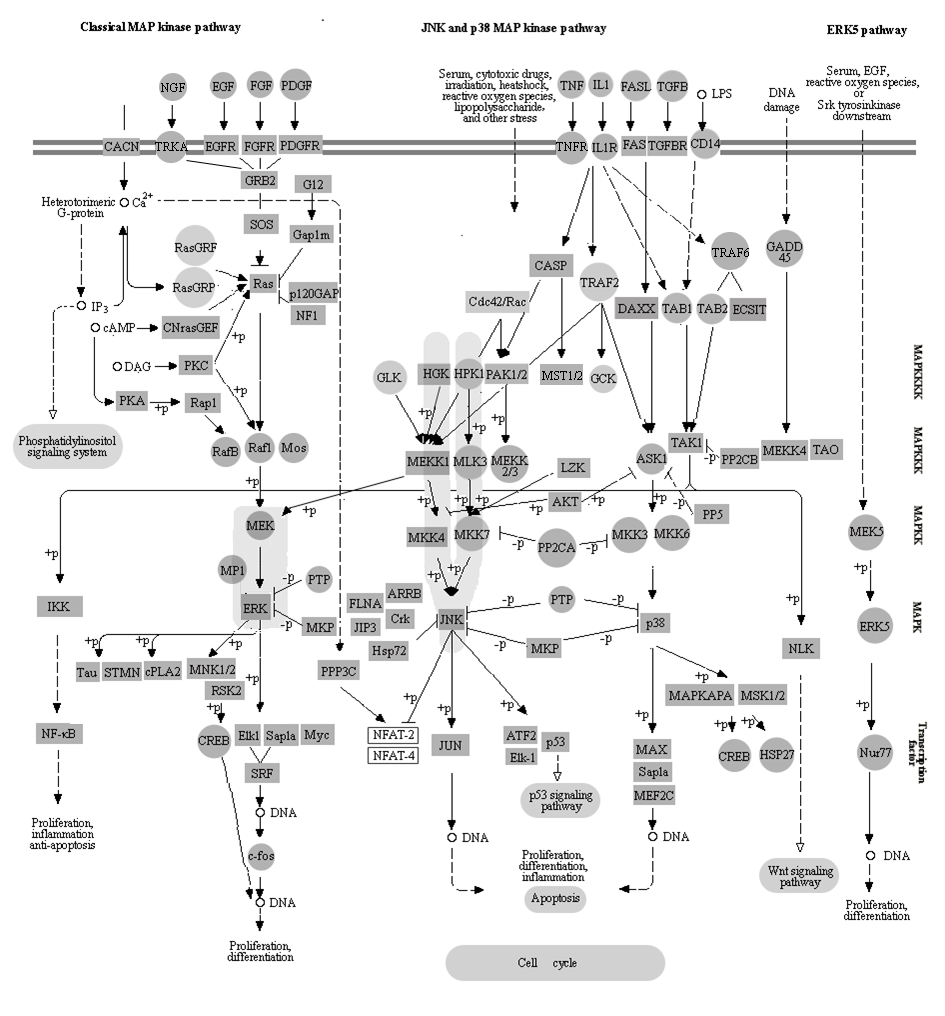

Supplement: Figure S4 — Putative MAPK pathway. Putative MAPK pathway of E. sinensis was constructed based on KEGG reference pathway. Proteins appearing in hepatopancreas of microbial challenged E. sinensis were represented in grey circle and absent proteins in grey square. However, most interactions have to be confirmed experimentally. (TIF) [file pone.0068233.s004.tif]
