# Supplementary material for: Transcriptome Analysis and Discovery of Genes Involved in Immune Pathways from Hepatopancreas of Microbial Challenged Mitten Crab Eriocheir sinensis
Source: PLoS One. 2013 Jul 17;8(7):e68233. doi: 10.1371/journal.pone.0068233 (PMC3714283; doi:10.1371/journal.pone.0068233)
Supplement: Table S1 — Genes and specific primers used for real-time PCR. (DOC) [file pone.0068233.s005.doc]

Table S1 Genes and specific primers used for real-time PCR

| Gene name | Primer sequence (5’-3’) | RPKM |
| --- | --- | --- |
| β-actin | F:GCATCCACGAGACCACTTACA R:CTCCTGCTTGCTGATCCACATC | - |
| ALF | F:GACCCTTTGCTGAATGCTTGA R:CTGCTCTACAATGTCGCCTGA | 1314.00 |
| ARG | F:ACATGATGGAGCGGCTGGGT R:GAGGGCGGTATTTGGAGGGA | 0.35 |
| Chi | F:CACGCGCCCATCAAGAAGT R:GTGCGTGAAGGGAACAAGTC | 62.73 |
| ChySP | F:AACGAAAACATCCAGGCAGT R:ACTGGCACACTGTAACACGGT | 1329.24 |
| cPLA2 | F:TGGGCCACGCAGAATAAAGT R:TTGTTGTTGACGAGGCAGAAGT | 23.18 |
| FLNA | F:AGCTGCTCATTGATGCCTCC R:TGTCCACCACCTTCACTTTCC | 74.39 |
| GPX | F:CAACGGGGCTGATGAAGAC R:AGGGTGGTAGCGGGTGTAT | 52.48 |
| GST | F:TCACCCGTCTTTGTTACAGG R:TTCGTCCAACAGCTCATAGA | 99.07 |
| HGF | F:CTTCCTCTGTGCTCTTGTCGG R:TGTCTGGGTCTGGGTGTTTG | 49.00 |
| HSP | F:CGGTGGCGGAGAAGTTGTC R:ATCTTGGAGGCGAGGGGA | 31.09 |
| IMD | F:AGGGATAAGGCTGCCGTTGT R:CGAGGGAAGTCTGCTGAAATGT | 1.62 |
| Int | F:GTGACGCCTAACGATGAGAA R:TGGGACAGAACAGCGAAGAT | 30.56 |
| LZM | F:GCAATGGTGATGGGAAGAT R:CACGTGTTGAACCTCTCGAA | 0.14 |
| ManR | F:AGGGAAAGGAGTCGGTGTG R:GCTAGTCATTGCCACACAGTTC | 2.89 |
| MasL | F:TGGGCATCGTCTTTTTCAGG R:TGGCAATGTCGTAGTCCTCGT | 0.72 |
| MBL | F:GCGAGGACAACAATGAGAGGT R:CGCAGATGGTCGGCTTTT | 5.66 |
| MKP | F:GGTTCTTCTCGCAGCTCATCG R:ACCGCTTGTCCTGCATGGC | 0.27 |
| PAK1 | F:AGCCGGTGATCAAGAAGAGG R:GAACGGGTGCTGAAGGAGTC | 29.76 |
| Pero | F:TCCAGGACCAGATCAACCCA R:CGATGCACGTGAAAGTCCAG | 1.93 |
| Rac1 | F:CTCCCATCGTGCTCGTTG R:GTACTTGGACAGGCGGTGA | 70.04 |
| ScaR | F:GTTTGAAGACGGTGTGGCT R:GGGTTGTTAGGAAAGCGATA | 0.35 |
| SOCS | F:CTAAAGTTGCCCCGAAGGTGA R:TGATACCAGCCACTTTCCTCC | 95.00 |
| Spa | F:CAGGGTGGAGAAGTGCATCAGT R:CGGGCTCGATCTTGTTGGT | 26.80 |
| Thy | F:CAGAAACAGCGGAAAAGG R:CCTGCTCAATGACTTCCTTAG | 1005.15 |
| TrxR | F:GGGTTTACATTTCCAGGTTG R:GACTCACTAGGATCGCTTTG | 145.27 |
| TrySP | F:CGCTTACGGAGAAAACGACA R:CAACCATAGCCCCAGGACA | 12079.92 |
| VEGFR | F:GGATGGTTACAGGATGGAGC R:CCTCTCCGACCTCTCTGAAG | 37.35 |

Notes: RPKM (Reads Per Kb per Million Reads) indicated abundance levels of expressed genes that were detected in Illumina sequencing. With a larger RPKM value, the expression level of sequenced gene was higher.
